# Orthodontics and Endodontics: clinical decision-making

**DOI:** 10.1590/2177-6709.25.3.020-029.oin

**Published:** 2020

**Authors:** Alberto Consolaro, Dario Augusto Oliveira Miranda, Renata Bianco Consolaro

**Affiliations:** 1 Faculdade de Odontologia de Bauru, Universidade de São Paulo (Bauru/SP, Brazil). Faculdade de Odontologia de Ribeirão Preto, Universidade de São Paulo (Ribeirão Preto/SP, Brazil).; Universidade de São Paulo, Faculdade de Odontologia de Ribeirão Preto, Universidade de São Paulo, Ribeirão Preto, SP, Brazil; 2 Universidade Estadual de Feira de Santana, Departamento de Saúde (Feira de Santana/BA, Brazil).; 3 Faculdades Adamantinenses Integradas (Adamantina/SP, Brazil).

**Keywords:** Orthodontics, Endodontics, Orthodontics-Endodontics, Tooth resorption

## Abstract

Endodontically treated teeth may be moved, as endodontic treatment is not a contraindication for orthodontic treatment. Apical periodontal repair begins when the periapical or pulp lesion has completely resolved. This may happen immediately after treatment if the filling material causes little or no irritation of periapical tissues, and particularly if the material is fully contained within the canal. When it leaks, a foreign body granuloma forms and persists for some months or indefinitely, depending on the composition of the filling material. Materials containing calcium hydroxide with no resin components undergo phagocytosis and disappear in some months, as macrophages gradually remove them. Materials containing resins, silicone, ionomers, zinc oxide-eugenol, bioceramics or gutta-percha remain in the site and induce the formation of foreign body granulomas. Although this does not preclude tooth movement, patients should be followed up every three months using periapical images to control the position of the material in relation to the tooth apex. “Pseudo” overfilling may be avoided if permanent filling is delayed until the time when orthodontic treatment is completed.

## RESUMO

Os dentes tratados endodonticamente podem ser movimentados, e não representam contraindicação para o tratamento ortodôntico. O reparo periodontal apical se inicia a partir do momento em que a causa da lesão pulpar e/ou periapical é eliminada. Isso ocorre de maneira imediata se o material obturador for pouco ou nada agressivo aos tecidos periapicais, especialmente se estiver limitado ao canal. Quando extravasado, haverá a formação de granuloma do tipo corpo estranho por alguns meses ou permanentemente, dependendo da composição do material obturador. Os materiais à base de hidróxido de cálcio sem componentes resinosos são fagocitáveis e, em alguns meses, desaparecem do local, pois os macrófagos irão gradativamente removê-los. Os materiais à base de resina, silicone, ionômero, óxido de zinco e eugenol, biocerâmicos e guta-percha permanecerão no local, induzindo granulomas periapicais do tipo corpo estranho, o que não inviabiliza a movimentação dentária, mas devem ser acompanhados a cada três meses com imagens periapicais, para um controle de sua relação espacial com o ápice dentário. A “pseudossobreobturação” pode ser evitada se a obturação definitiva for postergada para quando o tratamento ortodôntico terminar.

One of the issues that raises the most questions and uncertainties in the orthodontic and endodontic clinical practice is the time at which a tooth may be moved after the completion of an endodontic procedure. This study extrapolates experimental and clinical knowledge accumulated from studies and clinical cases^1^ ^-^ ^11^, particularly as there are no specific data about it in the relevant literature. 

## CHARACTERISTICS OF ORTHODONTIC FORCES

**Figure 1:**
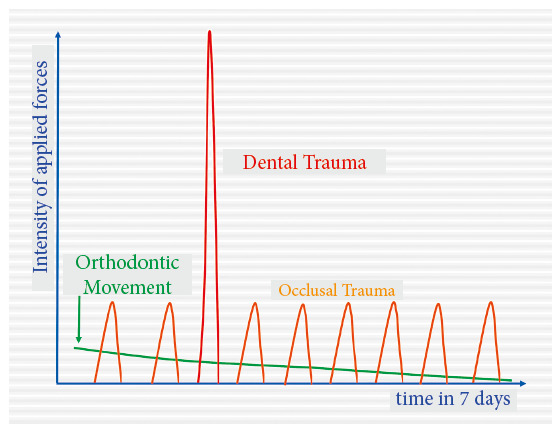
Comparison of intensity and duration of forces applied in orthodontic movement, occlusal trauma and dental trauma. Intensity peaks and dental trauma duration should receive special attention. Tissue changes are very different in each case.

Orthodontic forces, very differently from the forces of occlusion and dental trauma, are characteristically very light, dissipating and applied to tissues slowly. Even the orthodontic forces classified as heavy, severe or intense are lighter than those of dental and occlusal trauma. In summary (Fig 1), we may say that:

1. Occlusal forces are abrupt, have moderate intensity and short duration, but are very repetitive.
2. Forces of dental trauma, which are very intense, abrupt and have an extremely short duration, are highly damaging to periodontal and tooth tissues.
3. Orthodontic forces are not comparable with those of occlusal and dental trauma in all aspects and parameters. They are markedly lighter and dissipating, even though they might be called heavy or intense in an orthodontic context or environment.

## ORTHODONTIC MOVEMENT IS PRODUCED BY THE PERIODONTAL LIGAMENT

Orthodontic forces applied to the periodontal ligament slightly compress vessels and induce metabolic cellular stress by hypoxia. This stress is added to the mechanical cell stress that results from cytoskeletal deformation. In case of cellular stress, cells and tissues preserve their normal morphology under light microscopy, as this is not a disease or a case of tissue disorganization, but, rather, a differentiated stage of normal tissue with greater metabolic activity. In everyday life, tissues alternate from homeostasis to stress when performing their functions.

 Induction of cellular stress in the periodontal ligament by orthodontic forces promotes a greater release of mediators, particularly those associated with bone resorption, such as some cytokines and prostaglandins. The purpose of this tissue reaction is to enlarge the space for cells, which was reduced by periodontal tissue compression. As a result of that, teeth will be moved.

## ENDODONTIC TREATMENT DOES NOT CHANGE THE SURROUNDING PERIODONTAL ENVIRONMENT

Cementum covers the root surface and closes dentinal tubules externally. Periodontal fibers are inserted into the cementum. Cementum and dentin physiologically separate the pulp from the periodontal ligament so that pulp structures and functions are fully preserved, as demonstrated in several experimental studies that evaluated ligament changes induced by orthodontic movement. 

Periodontal tissues are microscopically normal when there are pulp changes or even necrosis. True endodontic-periodontic lesions are those that require both endodontic and periodontic treatment approaches, as they are lesions initially independent from each other, but that progress together along time. In other words, there is no efficient communication between the pulp chamber and the periodontium through the dentin and cementum wall, except in the case of lateral canals.

**Figure 2:**
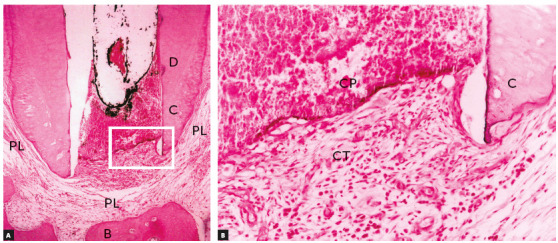
Tissue reaction after canal filling with cement plug (CP) where periodontal tissue organizes as new connective tissue (CT); discrete signs of mononuclear inflammatory infiltrate and discrete angiogenesis remain. Lateral aspect of periodontal ligament (PL) is normal. (D=dentin, C=cementum, B=bone, PL= periodontal ligament - HE, 10X and 40X). (Source: Esberard^9^, 1992).

When a root canal is filled, the material used does not affect the antigenic composition of the dentin or the cementum (Fig 2). It is not possible to say whether endodontic treatment increases or decreases orthodontically induced resorption. There is no methodological basis for such claims and, therefore, teeth that have been adequately treated endodontically should be considered orthodontically normal teeth in clinical practice.

## REPAIR OF PERIODONTIUM AND PERIAPICAL BONE LESIONS IN VITAL TOOTH PULPECTOMY

In the apical periodontal ligament continuous with the pulp at the margins of the cemental canal, repair in vital tooth pulpectomy begins as soon as filling is completed. In the interface between filling material and periodontal connective tissue, there is acute inflammation with edema and neutrophils. As there are no aggressors there, particularly no bacteria, this inflammation is reabsorbed and migrates to other areas (Fig 2) in 24-48 hours. 

**Figure 3:**
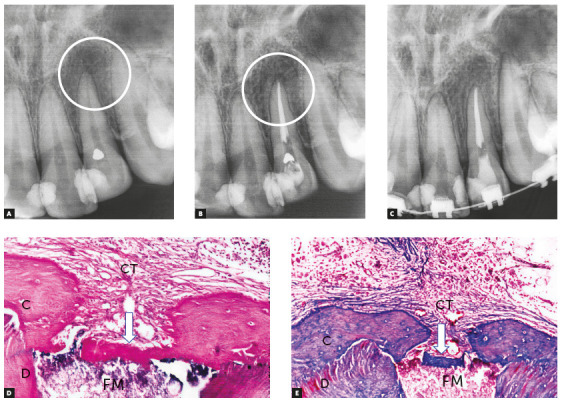
Chronic periapical lesion compatible with incipient immunogenic periapical granuloma (circle) associated with pulp necrosis and canal contamination. Tooth was moved orthodontically soon after that. In some weeks, periodontal ligament was repaired and restored with new connective tissue (CT); cementoid tissue may form at interface (arrows) depending on type of filling material (FM). (D=dentin, C=cementum - HE and MT, 10X. (Source: Esberard^9^, 1992).

After this short time, macrophages are predominant and participate actively in repair and the return to normality, as they phagocytize cellular debris that may have been produced by instrumentation. Depending on the type of filling material used, neighboring cementoblasts may proliferate and gradually migrate into the interface with adjacent tissues. There they may form cementoid material and result in the insertion of collagen fibers (Fig 3). This interface also has fibrous connective tissue without fiber insertions, but does not have any residual or persistent inflammation. 

**Figure 4:**
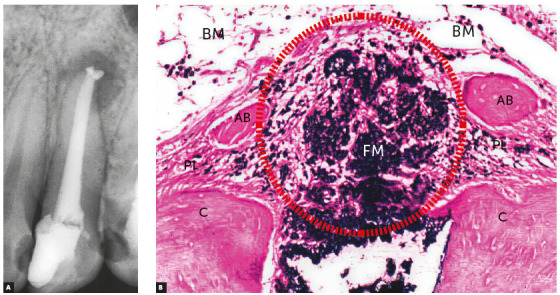
Chronic periapical lesion compatible with immunogenic periapical granuloma; during treatment, filling material (FM) leaked, which is seen microscopically as dark dots and clusters with macrophages around it and forming a periapical foreign body granuloma (circle). (D=dentin, C=cementum, AB=alveolar bone, BM=bone marrow, PL = periodontal ligament - HE, 10X. (Source: Leonardo^10^, 1992).

Macrophages can phagocytize, or try to phagocytize, some of the materials used for filling, gathering in their interfaces and forming a foreign body granuloma (Fig 4). If there are no microorganisms or sources of toxicity that may kill cells, this process remains there indefinitely, with no symptoms or detrimental effects to apical periodontal physiology. 

## REPAIR OF PERIODONTIUM AND APICAL BONE IN NON-VITAL TOOTH PULPECTOMY

After the endodontist eliminates the cause of pulp necrosis, particularly when it is induced by microbial agents, microbial products, such as enzymes and toxins, as well as lipopolysaccharides (LPS), are no longer expected to be found in periapical tissues. Endodontic treatments also eliminate microbial biofilm from the cemental canal and in the areas of root resorption of the cemental canal. This may result from mechanical action or the use of medications placed in the canal. 

**Figure 5:**
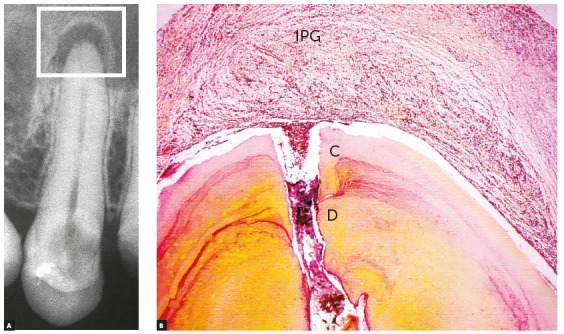
Chronic periapical lesion compatible with incipient immunogenic periapical granuloma (IPG) associated with pulp necrosis and canal contamination, as seen microscopically in B. (D=dentin, C=cementum - BB, 10X).

Periapical repair, which includes the bone and cementum, initiates as soon as aggressors are eliminated (Figs 3 and 5). Mean microbial survival time is 20 minutes for each generation. Therefore, between 24-48 hours, macrophages complete the phagocytosis of tissue and microbial residues. Exudate absorption is being completed by venous and lymphatic vessels. Therefore, cytotoxic microbial products, such as toxins, enzymes and LPS, are about to be fully eliminated.

However, this may not occur in all cases, as there may be failures even when the treatment is adequate. In some cases, microbial biofilm forms on the external surfaces of the apex, or even in more profound areas of external inflammatory resorptions.

## REPAIR OF PERIODONTIUM AND APICAL BONE IN THE PULPECTOMY OF NON-VITAL TEETH WITH CHRONIC PERIAPICAL LESION

In addition to teeth with infected pulp necrosis still restricted to the root canal, there are also cases of infected pulp necrosis and chronic periapical lesions, particularly immunogenic periapical granulomas (Figs 3, 4 and 5). In practically all teeth with a chronic immunogenic periapical granuloma, external inflammatory resorptions are somehow intense, which makes it difficult to remove bacteria from within the dentinal tubules or from the surface irregularities in the area.

In teeth with chronic periapical lesions, microbial biofilm out of the cemental canal is more frequent and larger. There are also more free bacteria and microbial clusters inside the lesion. This complicates instrumentation and bacterial elimination by canal medications. 

Such complication may explain the existence of a small percentage of these cases, which accounted for about 10% to 20% of a sample of teeth with pulp necrosis and chronic periapical lesions analyzed. In these cases, periapical lesions may persist and stabilize or grow along time, in which case they require endodontic retreatment or surgery. There will be no repair because of the persistence of aggressors, that is, bacteria and their products, particularly in microbial biofilm.

## REPAIR OF PERIODONTIUM AND PERIAPICAL BONE IN ASEPTIC PULP NECROSIS

**Figure 6:**
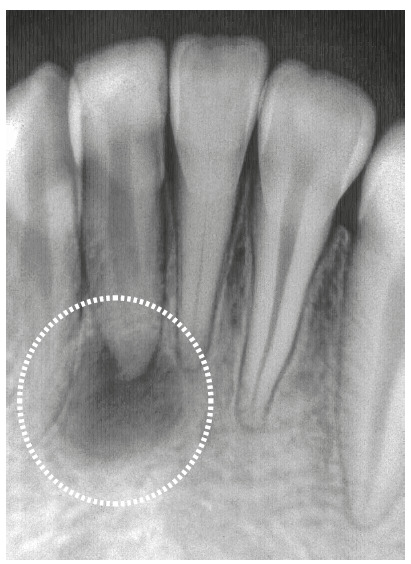
Chronic periapical lesion compatible with periapical granuloma associated with aseptic pulp necrosis caused by products of tissue necrosis.

No microorganisms are involved in aseptic pulp necrosis (Fig. 6), except when this necrosis lasts longer than one year, in which case bacteria may reach that site via blood or anachoresis. 

Adequate endodontic treatment of these cases usually leads to periapical and bone repair similar to that found in cases of vital tooth pulpectomy. After adequate root filling, a rapid acute inflammatory response initiates in 24-48 hours, soon leading to repair, because the main aggressor was in the products of aseptic pulp necrosis, which are considerably less aggressive to tissues and are previously eliminated. 

## ORTHODONTIC FORCES DO NOT CHANGE THE MICROBIOTA OF CANALS AND CHRONIC PERIAPICAL LESIONS: WHEN SHOULD MOVEMENT BEGIN?

Orthodontic forces are light, dissipating and not applied abruptly (Fig 1). These forces should partially compress the periodontal ligament, at a thickness of 0.25 mm and 50% of the volume taken up by blood vessels. They should be light so that they produce tooth movement effectively. If forces are excessive, the connective tissue of the ligament hyalinizes, and osteoclasts and other cells do not resorb bone, nor, therefore, move teeth. This means that excessive force will not be effective.

Orthodontic forces are so light that they do not affect the tissue and cell phenomena that are proper to apical and periapical repair. Cell migration and proliferation are not affected, nor are angiogenesis or collagen synthesis. In addition, orthodontic forces do not affect the microbiota in the canal, the cemental canal and external apical areas. 

**Figure 7:**
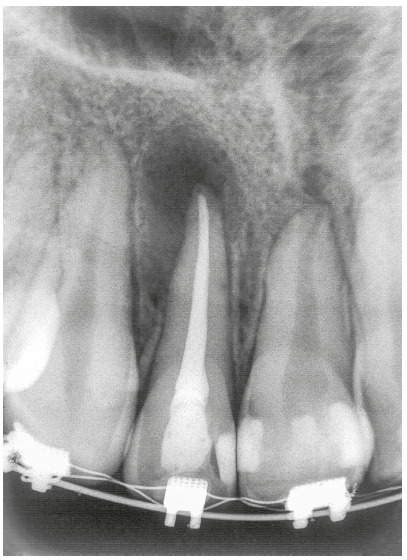
Chronic periapical lesion compatible with immunogenic periapical granuloma treated endodontically in tooth beginning to be moved orthodontically.

**Figure 8:**
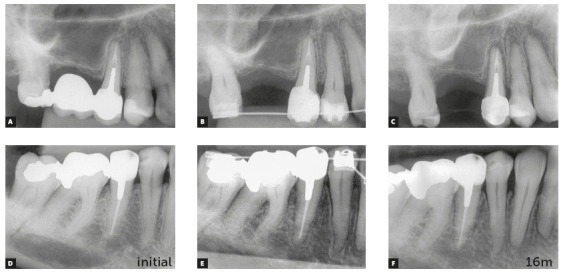
Premolars endodontically treated and orthodontically moved 16 months after completion of orthodontic treatment. (Case reported by Antônio Geraldo de Oliveira, Varginha, Brazil).

In the past, orthodontic movement of teeth treated endodontically was contraindicated, but today these teeth should be considered normal for orthodontic purposes. No evidence in the literature indicates that tooth movement may be detrimental to the repair of apical and periapical tissues, which includes bone. Such outcomes occur regardless of the time waited before applying orthodontic forces (Figs 7 and 8).

These explanations and the reasoning presented before may allow us to confidently conclude that teeth may be moved soon after endodontic treatment without affecting the repair of apical and periapical tissues (Figs 7 and 8). Treatment may be delayed for 7 to 15, or even 30 days, so that tissues reorganize. In fact, not even this waiting time is necessary. The same applies equally to teeth with or without chronic periapical lesions. These cases should be followed up with periapical images obtained every three months.

The relationship between the endodontist and the orthodontist should be constructive and collaborative. If orthodontic movement is applied immediately and endodontic treatment fails, failure should be assigned to the technical limitations of all specialties, including endodontics, and not to the fact that the tooth was moved. Orthodontic treatment should not be included as an explanation in studies about the reasons of endodontic failures, as orthodontic movement does not produce this type of failure. 

## “PSEUDO” OVERFILLING: A MOMENT TO AVOID

What is the best time to fill a canal: when the patient is beginning or in the middle of an orthodontic treatment? The ideal time is when the canal is ready to receive permanent filling, but not before that. A temporary filling should be used while the orthodontic treatment progresses as usual, and this decision should be jointly made by the orthodontist, the endodontist and the patient.

**Figure 9:**
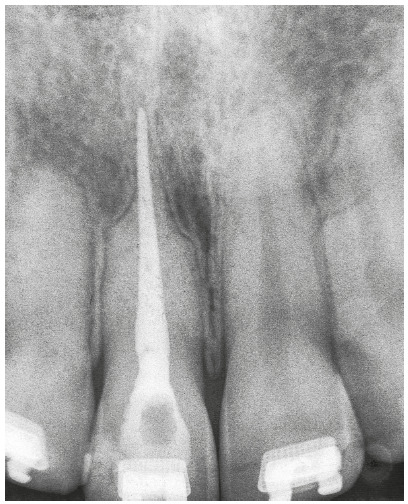
“Pseudo” overfilling associated with external inflammatory apical resorption that may be avoided by delaying permanent canal filling if patient is followed up and there is a collaborative relationship between orthodontist and endodontist. (Case reported by Armelindo Roldi, Vitória, Brazil).

**Figure 10:**
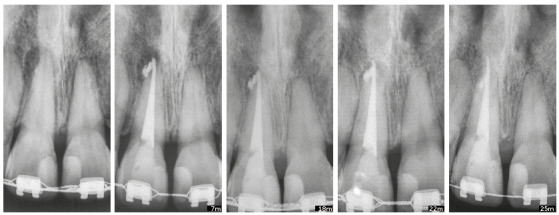
Chronic periapical lesion compatible with incipient immunogenic periapical granuloma associated with pulp necrosis. Filling material leaked, and follow up confirmed that it was not aggressive, even 25 months after orthodontic movement. Attention is drawn to "pseudo” overfilling by gutta-percha cone and similar degree of external inflammatory apical resorption in adjacent tooth. (Case reported by Vanessa Bernardini Maldonado, Ribeirão Preto, Brazil).

Teeth that have been moved may occasionally present with external inflammatory apical resorptions. If this is the case, and the tooth has permanent filling in the canal, the outcome may be a "pseudo" overfilling, in which cones and filling cements are found beyond the original apical end (Figs 9 and 10).

If the filling is temporary, arrangements for the permanent filling may be made as soon as the orthodontic treatment is completed, and then be contained within current limits of the tooth apex. “Pseudo” overfilling is, thus, avoided. This will be possible only if there is a collaborative and constructive relationship between the orthodontist and the endodontist, and when the patient has the necessary information and agrees with the decision.

## APICAL LEAKAGE AND ORTHODONTIC MOVEMENT

Another point to be analyzed in the association of endodontic and orthodontic treatments is the possibility of material leaks into the apical region.

Calcium hydroxide filling materials that leak into the apical region induce the formation of foreign body granulomas around them. However, macrophages can phagocytize their particles, and there will be no more opaque structures on images of periapical tissues six to twelve months later. This non-aggressive leakage is reabsorbed and does not preclude tooth movement. Therefore, it will not have any significant consequences after tooth movement is completed (Fig. 10).

Fillings with glass ionomer, bioceramics, zinc oxide-eugenol cements, resin cements and gutta-percha cones are not phagocytized or removed by macrophages and osteoclasts. Once they leak out, they will induce the formation of foreign body granulomas around that area, and will remain there for an indefinite amount of time, precluding the complete repair of apical and periapical tissues. Imaging studies at regular intervals may provide information to control this clinical condition without inducing an immune response if the case is asymptomatic or has only a few symptoms. One frequent symptom is sometimes described as a low-intensity pricking pain during mastication, as the apex applies pressure to the material against the bone (Fig 4).

If teeth with accidental leakage of material into periapical tissues have to be moved, orthodontic treatment may be confidently considered. However, periapical images should be obtained every three months to control the position of material in relation to the apex (Fig 10). This is recommended because there may be a more localized apical resorption, specifically associated with the material when the tooth moves towards the material itself. However, this does not have serious consequences for periodontal support.

## FINAL CONSIDERATIONS

1. Teeth that received adequate endodontic treatment may be moved, as endodontic treatment is not a contraindication for orthodontic treatment.
2. Apical periodontal repair begins when the cause of periapical or pulp lesion has been eliminated. This occurs immediately after the filling material becomes little or not aggressive to periapical tissues, and particularly if the material is fully contained within the canal.
3. When the filling material leaks into the apical area, a foreign body granuloma forms and then persists for some months or indefinitely, depending on its composition.
4. Materials containing calcium hydroxide with no resin components undergo phagocytosis and disappear from the site in some months, as macrophages gradually remove them.
5. Materials containing resins, silicone, ionomers and zinc oxide-eugenol, as well as bioceramics and gutta-percha, remain in the site and induce the formation of foreign body granulomas. This does not preclude tooth movement, but the patient should be followed up every three months using periapical images to control the position of granulomas in relation to the tooth apex.
6. “Pseudo” overfilling may be avoided if permanent filling is delayed until the endodontic treatment is completed.

A better understanding of these cases may be achieved from the comparison of images of a large number of teeth treated endodontically and moved orthodontically after they have been grouped according to their variables. Observations made in clinical or animal studies may be confirmed statistically, as well as in clinical practice and through random findings during consultancy activities.
